# Optogenetic self-stimulation in the nucleus accumbens: D1 reward versus D2 ambivalence

**DOI:** 10.1371/journal.pone.0207694

**Published:** 2018-11-29

**Authors:** Shannon L. Cole, Mike J. F. Robinson, Kent C. Berridge

**Affiliations:** 1 Department of Anatomy and Neurobiology, University of Maryland School of Medicine, Baltimore, Maryland, United States of America; 2 Department of Psychology, Wesleyan University, Middletown, Connecticut, United States of America; 3 Department of Psychology, University of Michigan, Ann Abor, Michigan, United States of America; University of Leicester, UNITED KINGDOM

## Abstract

The nucleus accumbens (NAc) contains multiple subpopulations of medium spiny neurons (MSNs). One subpopulation expresses D1-type dopamine receptors, another expresses D2-type receptors, and a third expresses both. The relative roles in NAc of D1 neurons versus D2 neurons in appetitive motivation were assessed here. Specifically, we asked whether D1-Cre mice would instrumentally seek optogenetic self-stimulation specifically targeted at D1 MSNs in NAc, and similarly if D2-Cre mice would self-stimulate D2 neurons in NAc. Mice were implanted with Cre-targeted channelrhodopsin (ChR2) virus and optic fibers in NAc. Subsequently, mice could earn brief NAc laser illuminations by actively touching a metal spout in one task, or by going to a particular location in a separate task. Results indicated that D1 neuronal excitation in NAc supported intense self-stimulation in both tasks. D1-Cre mice earned hundreds to thousands of spout-touches per half-hour session, and also sought out locations that delivered NAc laser to excite D1 MSNs. By comparison, D2 ChR2 mice showed lower but still positive levels of self-stimulation in the spout-touch task, earning dozens to hundreds of NAc laser illuminations. However, in the location task, D2 mice failed to show positive self-stimulation. If anything, a few D2 individuals gradually avoided the laser location. Brain-wide measures indicated that D1 and D2 stimulations in NAc recruited heavily overlapping patterns of Fos activation in distant limbic structures. These results confirm that excitation of D1 MSNs in NAc supports strong incentive motivation to self-stimulate. They also suggest that excitation of D2 neurons in NAc supports self-stimulation under some conditions, but fails under others and possibly may even shift to negative avoidance.

## Introduction

The nucleus accumbens (NAc) is important to appetitive motivation for diverse rewards, which range from food, sex, addictive drugs and brain self-stimulation in animals and humans, to more abstract rewards such as music at least in humans [1–11].

Within the NAc are distinct subpopulations of GABAergic medium spiny neurons (MSNs), which differ in their expression of dopamine receptors (D1-type versus D2-type), and in connectivity to other structures. From NAc, D1 MSNs project ‘directly’ to the midbrain ventral tegmental area (VTA), whereas D2 MSNs instead project only ‘indirectly’ from NAc to targets such as ventral pallidum (VP) and lateral hypothalamus (LH) in basal forebrain. To that extent, NAc connectivity resembles that of neostriatum, where D1 MSNs project ‘directly’ to midbrain targets such as substantia nigra, while D2 MSNs project ‘indirectly’ to forebrain targets such as globus pallidus [12,13]]. However, NAc D1 MSNs also send ‘indirect’ projections to VP and LH, similarly to NAc D2 MSNs, which dilutes the NAc distinction between ‘direct’ versus ‘indirect’ outputs [13–16] . In addition, a third group of up to 30% of MSNs in NAc shell are reported to co-express both D1 and D2 receptors on the same neuron, which also likely project to indirect VP and LH targets [17,18]. Finally, acetylcholine neurons in NAc and neostriatum may also express D2 receptors [19,20].

What are the respective roles of D1 neurons vs D2 neurons of NAc in reward motivation? In dorsal neostriatum, D1 MSN excitation is reported to support optogenetic laser self-stimulation in mice, which instrumentally work to turn on illumination [21]. By contrast, D2 neuronal stimulation is avoided in dorsal neostriatum [21], though perhaps not in ventrolateral neostriatum [22,23]. In NAc, substantial evidence also supports a role for D1 MSNs in positive motivation for reward. For example, optogenetic D1 MSN stimulation in NAc enhances drug-induced conditioned place preferences [24,25]. Similarly, NAc pharmacological D1 receptor stimulation in D1 MSNs promotes incentive motivation to pursue or consume food or drug rewards [26–28].

In NAc, D2 receptor activation has been often oppositely linked to suppression of appetitive motivation or reward, such as measured by conditioned place preference [24,25], and even to generation of negatively-valenced avoidance or defensive behaviors, including fearful anti-predator responses [29–33]]. However, other studies have indicated positive appetitive motivation functions for D2 neurons in NAc [34–37].

The present study aimed to more directly compare motivation roles of D1 neurons vs D2 neurons in NAc in optogenetic self-stimulation that selectively excites either one or the other NAc population. Two self-stimulation tasks were used to compare D1-Cre versus D2-Cre transgenic mice that could earn laser excitations of Cre-targeted channelrhodopsin (ChR2) expressed in either D1 or D2 types of NAc neurons. Our findings indicate that D1 MSN excitation supports rapid, robust, and intense NAc laser self-stimulation, in both an active response spout-touch task and a relatively passive place-based self-administration task. By comparison, NAc excitation of D2 neurons produced weak positive self-stimulation in the active-touch task, but did not support self-stimulation in ChR2-expressing D2-Cre mice in the place-based task, and, if anything, eventually produced mild place avoidance for a few D2-Cre individuals.

## Methods

### Overview

Two independent self-stimulation procedures were used to allow mice to earn brief NAc laser stimulations as reward. First, an active spout-touch self-stimulation procedure allowed mice to earn brief 1-sec laser pulses in NAc each time they touched a particular metal spout (referred to as laser-spout) that protruded into the chamber. Another spout was also present, but earned nothing when touched, and served as a control stimulus for comparison. Second, a separate place-based self-stimulation task allowed mice to earn series of laser pulses by entering a particular corner of a 4-corner chamber and remaining there; this task was based on the original Olds and Milner procedure that discovered deep brain self-stimulation when a rat went to a particular location to activate its electrode [38]]. After the location task, some mice were also retested on the spout task to reconfirm their initial results.

### Subjects

BAC transgenic mice on a C57Bl/6 background (n = 59) were obtained from NINDS/GENSAT (www.gensat.org) from Rockefeller University/NIH/NIMH, and maintained on a 12-hour reverse light-dark cycle with food and water *ad libitum*. These included 33 D1-Cre mice (12 male, 21 female; strain: B6.FVB (Cg)-Tg (Drd1a-cre) EY262Gsat/Mmucd), and 26 D2-Cre mice (12 male, 14 female; strain: B6.FVB (Cg)-Tg (Drd2-cre) ER44Gsat/Mmucd).

D1-Cre females and males, and D2-Cre females and males were randomly assigned to receive NAc infection with either a channelrhodopsin virus (AAV5-DIO-ChR2-EYFP) to be an optogenetic group, or an optically-inactive virus to be an EYFP-only control group that lacked the ChR2 gene (AAV5-DIO-EYFP). This created four Cre/Virus groups for all following experiments: 1) ChR2 D1-Cre [n = 14 total (4 male, 10 female)]; 2) ChR2 D2-Cre [n = 14 total (7 male, 7 female)]; 3) EYFP D1-Cre control [n = 19 total: (8 male, 11 female)]; 4) EYFP D2-Cre control [n = 12 total: (5 male, 7 female)]. Male and female mice were housed separately, and test chambers were cleaned after each mouse was tested to avoid pheromone contamination. All animal protocols were in accordance with the National Institutes of Health Guide for Care and use of Laboratory Animals and approved by the University Committee on the Use and Care of Animals at the University of Michigan.

### Viral vectors

A DIO Cre-dependent ChR2 Adeno-associated virus (AAV) was used to infect Cre-expressing cells (vectors-double loxP-flanked inverted (DIO)—channelrhodopsin 2 (ChR2)—enhanced yellow fluorescent protein (EYFP) (AAV5-DIO-ChR2-EYFP; purchased from the University of North Carolina Vector Core with MTA by courtesy of Karl Deisseroth and Stanford University).

### Surgery

Mice were anesthetized with isoflurane gas (4–5% induction, 1–2% maintenance), placed in a stereotaxic instrument (David Kopf Instruments), and the skull surface was exposed. Bilateral microinjections of virus (0.5 μl per side; 0.1 μl/min) into NAc were targeted at the medial shell and medial core. Either ChR2 virus (AAV5-DIO-ChR2-EYFP) or an optically-inactive control virus (AAV5-DIO-EYFP) (0.5 μl) was delivered via 28 gauge syringe over 5 min, and left unmoved for 10 minutes to allow for viral diffusion. Stereotaxic coordinates for virus microinjections centered around AP +1.42 to +1.32; ML +/- 1.5; DV– 4.78; injectors were angled at 12.29 lateral degrees to avoid ventricles and permit space for bilateral fiber implants. NAc sites were staggered slightly across individual mice to nearly fill the entire medial shell as a group and include some penetrations in core (AP coordinates range from +1.42 to +1.32), but within a mouse bilateral sites were kept as symmetrically identical as possible.

In the same surgery, optic fibers (6 mm long; 0.220μm core; confirmed to exceed 85% light efficiency prior to surgery) were bilaterally implanted in NAc approximately 0.3 mm above each site of virus injection (AP + 1.42 to +1.32; ML +/- 1.5; DV– 4.48; 12.29 degree lateral angle). Optic fibers were anchored to the skull using surgical screws and dental acrylic. Mice were allowed at least 4 weeks after surgery for incubation and virus expression before behavioral tests began.

### Experiment 1: Laser self-administration-spout touch tasks

NAc self-stimulation was tested first using a spout-touch self-administration task, in which active touches of a designated empty metal drinking-spout could earn phasic 1-sec illuminations of NAc laser. Optic fibers were attached through an FC/PC adaptor to a 473 nm blue DPSS laser (OEM Laser Systems). Two empty metal spouts (lickometer touch-capacitive detectors) protruded through the wall of the 8x10x5cm chamber (MedAssociates Inc.), placed approximately 5 cm apart(See S1 File for example).

Active touches of one arbitrarily-designated spout (laser spout) delivered a brief 1-sec bin of laser illumination activated by an Arduino control board (Arduino Hardware), and accompanied by a distinctive auditory cue that served as a sensory label for the laser-delivering spout (either white noise or tone, 1 sec). Touching the other non-laser spout produced no laser, but did produce the different auditory cue as a distinctive marker, and touches on it served merely as a control measure of generalization, exploration and general motor activity (no-laser spout).

In an initial screening wave of mice (n = 6 D1 ChR2; n = 5 D2 ChR2; n = 7 D1 EYFP & n = 6 D2 EYFP), we compared the relative effectiveness of constant laser versus pulsed 25 Hz laser in 1-sec illumination bins during 30 min sessions, at intensities of either 0.1, 1.0 or 10 mW. One spout delivered laser and was reliably paired either with white noise or with a pure tone as auditory marker of laser-illumination. The other spout delivered no laser, but touches produced the other sound; spout/noise-tone assignments were balanced across mice. Optic fiber light transmission at the end of the output optic cable was confirmed each day (Laser Check Photometer, Clairvoyance Inc.), and cranial fiber implants had been tested for 85% efficacy prior to surgery. Individual D1 ChR2 mice or D2 ChR2 mice were excluded from being considered self-stimulators if they failed to meet both criteria of 1) at least 1 session of 20 contacts on the laser-delivering spout and 2) a 2:1 ratio of laser-paired versus non-laser paired contacts on at least one test day. Constant illumination and 25Hz pulses of laser stimulation were compared at three different laser intensities: 0.1, 1.0 and 10 mW. From results of this preliminary comparison, 1mW constant illumination was found to produce reliable self-stimulation, and utilized for all further spout self-stimulation experiments.

#### Location-tracking across 9 days

Laser at 1.0 mW intensity and the constant-illumination 1-sec parameters produced moderate levels of NAc self-stimulation in the initial screening test, and was focused on here for a more thorough comparison of NAc D1 vs D2 self-stimulation, in a 9 day test in which laser location sometimes moved (n = 9 D1 ChR2 mice; n = 9 D2 ChR2; n = 6 D1 EYFP and n = 6 D2 EYFP). Constant laser illumination at low intensity was chosen also because it has been suggested to facilitate more naturalistic or endogenous patterns of neuronal firing than arbitrarily-pulsed stimulation frequencies such as 25 Hz [39,40]. For example, constant stimulation at 1 mW is reported to preserve firing waveform characteristics in both D1 and D2-expressing striatal neurons, similar to firing waveforms of the same neurons during control periods without laser stimulation [39].

Additionally, we wished to assess whether self-stimulations truly motivated in the sense of being instrumental actions that were directed flexibly and specifically aimed at obtaining NAc ChR2 laser excitations, or instead if spout-touches were merely being stamped-in rigid stimulus-response habits or simply repeated as a mere motor reaction to an immediately prior NAc stimulation. Therefore, during the 9-daily sessions we shifted the location of the laser spout three times (a shift every 3 days) to test whether mice would flexibly track the source of NAc laser as spouts moved. The location of the two spouts were fixed across the first three days of testing (Days 1–3; active/inactive locations were counter-balanced across mice). On Day 4, the active laser spout and inactive spout were both moved to new positions on the opposite wall, and then kept stable over Days 4–6. On Day 7, both spouts were moved back to the original wall, but reversed from their original positions on Days 1–3, so that the laser spout now occupied the former no-laser spout position and vice versa, and kept in their new positions over Days 7–9. This presented the mouse with a 3^rd^ new location for the active spout, which was exactly opposite to its original location.

#### Laser-extinction test

Finally, on the 10^th^ day, an extinction session with no laser was given to further assess if self-stimulation behavior became habitual or aimed at conditioned reinforcement, or instead remained flexible and dependent on NAc laser activation. In the extinction session, the laser reinforcement was discontinued, though touching each spout still produced its associated auditory cue.

### Experiment 2: Location-based self-stimulation task

A second location-based self-stimulation task was conducted subsequently, which allowed NAc laser stimulations to be earned more passively by simply entering or remaining in a particular corner location in a 4-corner chamber. The center of the 38x38cm floor of the chamber was occluded by a large cylinder (20cm diameter plexiglass), so the mouse could circumnavigate only along the outer periphery and among the four corners of the chamber, and the floor of the chamber also contained bedding. Within the laser-delivering corner, any movement triggered an infrared motion detector that delivered a 1-sec laser constant illumination (1mW, constant) per movement during a 30-min session. Each corner had its own motion detector (Visonic), and MATLAB software was utilized to compile entries and time spent within each of four corners. One corner was arbitrarily designated as laser-delivering on the first day (corner assignment balanced across mice; laser corner changed on subsequent days as described below). Each corner had its own infrared motion-detector that detected entries and recorded time spent in that corner. Triggering the detector for the laser corner activated the laser illumination, and duration of time spent in the corner was registered. Laser stimulations were earned on entry into the laser corner and by every further movement detected while the mouse remained within that corner. Laser immediately ceased when the mouse left the designated corner. Entries and time spent in the other three corners were also monitored but did not produce laser illuminations. On the second test day, the laser corner designation was shifted to a different corner, diagonally opposite the Day 1 laser-corner. On the third day, the active corner designation was arbitrarily shifted to a new location in one of the remaining two corners (i.e., moved clockwise or counter-clockwise).

This location-based procedure can assess negative *avoidance* of the laser-corner, as well as positive preference. Avoidance would be evident by a mouse not entering or more quickly leaving the laser-delivering corner compared to alternative corners. Behavior was also videotaped each day for subsequent video analysis and scored offline for duration (seconds) engaged in the following behaviors: burrowing (submerging head and using bilateral forepaw movements to throw bedding backwards), defensive treading (throwing bedding forward via alternating unilateral forepaw movements), rearing (elevating body and head together on hindpaws so that forepaws rose >1cm above floor), locomotion (seconds of continuous forward movement), and immobility. Locomotion was assessed both throughout the entire session, and also specifically during and 2-sec after laser illuminations.

#### Histological analyses of virus expression, local Fos plumes, and distant Fos activations

Immediately before euthanasia, a standardized dose of laser stimulations was passively delivered to all mice in order to 1) generate local Fos plumes of neuronal Fos activation immediately surrounding the fiber tip in NAc, and 2) to potentially recruit distant Fos activations in other brain structures that would reflect functional connectivity patterns for D1 vs D2 circuitry. Beginning 90 minutes prior to euthanasia and perfusion, each mouse was put into a self-administration spout chamber and given 1s bursts of 1mW laser stimulation every 10 seconds for 30 min. Laser stimulation was not contingent on any behavior prior to perfusion, so as to ensure equal laser exposure for D1-Cre and D2-Cre mice. All mice were then deeply anesthetized with an overdose of sodium pentobarbital, transcardially perfused, and brains were removed and analyzed for Fos plumes, and for distant Fos recruited in other limbic structures. Briefly, brains were stored in 4% paraformaldehyde for 1-day post-perfusion, and then soaked in a 30% sucrose solution for 2 days prior to slicing. 40 micron slices were blocked in 5% normal donkey serum/0.2% Triton-X solution for 30 min before being incubated for 24h in a polyclonal rabbit anti-c-fos IgG primary antibody (Santa Cruz Biotechnology; 1:1000 dilution; lot #K0415, RRID: AB_2106783), followed 1 day later by Alexa Fluor 594 donkey anti-rabbit IgG secondary antibody (Life Technologies; 3:1000 dilution; lot #1668652, RRID: AB_141637). Sections were mounted, air-dried, and cover slipped with ProLong Gold anti-fade re-agent (Invitrogen). Fos plumes, or local neurons expressing Fos surrounding an optic fiber tip, were counted using a grid with 8 arms emanating from the fiber tip, each arm containing consecutive 50-micron boxes, which were overlain until Fos decreased to baseline levels or reached the medial hemisphere. This analysis permitted calculation of the relative increase or decrease within a set area from the fiber optic tip, yielding a Fos density plume. Distributed Fos was calculated by counting Fos within selected regions within a 600x800-micron sampling box for each brain structure. A constant-size sampling box was used to allow comparison between structures without confound from them being of different sizes. Viral infection was also measured by placing a similar grid overlay and measuring EYFP fluorescence in 50-micron increments until levels fell to baseline levels.

All images were taken using a Leica DM6000 B/M microscope and Surveyor 8.0 scanning software for tiling at 10x magnification (Objective: Leica HC PL APOL 10x/0.40; CS/0.17/1A). Fos was image using a TX2 size K filter system, and EYFP was imaged with a L5-FITC size K filter system purchased through Leica microscopy.

### Statistics

For initial statistical analyses, parametric 2-way ANOVAs were performed to determine main and interaction effects. If initial analyses were significant, subsequent non-parametric 1-way ANOVAs were applied: either Friedman’s for within-subject repeated-measures or Kruskal-Wallis ANOVAs for between-subject one-factor comparisons. For significant factors, further non-parametric pairwise Mann-Whitney or Wilcoxon tests were used as appropriate for subsequent paired comparisons. Spearman’s Rho was used for all correlational analyses. Shapiro-Wilks tests were used to detect non-normal distribution for classification of data as parametric or non-parametric. For all analyses, the significance level was set at p = .05; two-tailed. Effect sizes for pairwise comparisons were calculated using the following formula: $r=\frac{Z}{\sqrt{N1+N2}}$. Average self-stimulation rates of the two control groups (D1 EYFP and D2 EYFP) within the spout-self stimulation and operant place preference tasks were statistically similar (D1 EYFP: 9 (SEM± 4); D2 EYFP: 13 (SEM±3); Kruskal-Wallis: *X*^*2*^ = 2.01, p = .156) and so were combined into a single EYFP control group for comparisons.

## Results

### Overall 9-day active-spout self-stimulation

Across 9 days, a laser spout (1 sec constant-illumination laser at 1 mW intensity) and non-spout were available in all session. The location of the laser spout was shifted twice: once on Day 4 to a new location and again on Day 7 to a third location. The question was whether mice would self-stimulate, and flexibly track their moving source of laser activation to demonstrate motivated laser-seeking.

An initial two-way ANOVA indicated both that laser delivery was a factor driving spout touches, and that D1-Cre vs D2-Cre mice differed in number of spout-touches (Two-Way ANOVA: Laser vs non-laser x D1 vs D2: *F*_(2, 192)_ = 4.247, *p* = .026; Main effect laser vs non-laser: *F*_(1,24)_ = 5.770, *p* = .024; Main Effect D1 vs D2: *F*_(2,24)_ = 4.424, *p* = .023). However, female and male mice within each group did not appear to differ in self-stimulation: sex was not a significant factor, nor did sex interact significantly with other factors (Two-way ANOVA: Sex x Laser vs non-laser: *F*_(1,192)_ = 2.036, *p* = .167; Main effect of Sex: *F*_(1,24)_ = 1.867, *p* = .185; D1 vs D2 x Sex: *F*_(1,24)_ = 1.930 *p* = .167; Day x Sex: *F*_(8,192)_ = 1.492, *p* = .162). As no sex differences were detected in self-stimulation behavior here, males and females were subsequently combined within each condition for all further analyses Also, as distributions were detected to not be normal, non-parametric tests were used for subsequent analyses (D1 ChR2 laser vs non-laser: Shapiro-Wilk, W = .585, df = 81, p < .001; D2 ChR2 laser vs non-laser: W = .705, df = 81, p < .001).

### D1-Cre patterns of spout self-stimulation

**Fig 1:**
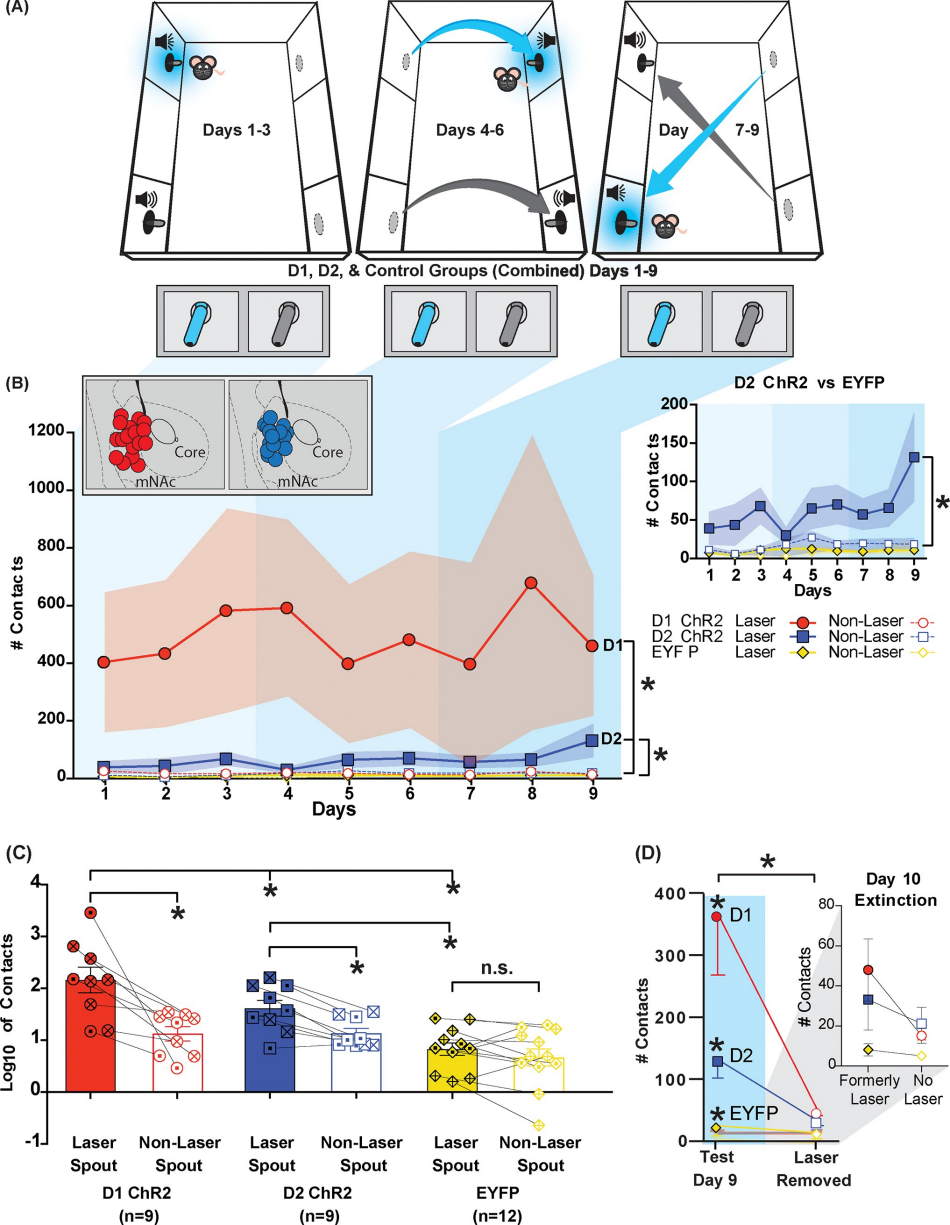
Spout self-stimulation totals for extended 9-day spout-task (1 mW, constant illumination, 1s): Strong D1 and weak D2 self-stimulation. **(A)** Laser-spout and non-laser spout locations are shown, with novel position shifts on Day 4 and Day 7. **(B)** Self-stimulation totals for D1 mice (red fill circles = laser; red surround circles = non-laser) or D2 mice (blue fill squares = laser; blue surround squares = non-laser) with ChR2 and EYFP control mice (yellow fill diamonds = laser; yellow surround diamonds = non-laser). Each point shows mean contacts per day for D1, D2 and EYFP groups, across all 9 days. Vertical shading delineates the responding during each 3-day laser spout position. Insert **(top right)** shows data for D2 ChR2 mice and EYFP control mice. D1 mice developed robust self-stimulation and bias for the laser-paired spout, making >500 times on average and at a ~27:1 ratio for laser-spout vs non-laser spout. D2 mice also developed preferences for the laser-paired spout (blue; see insert) though more weakly, self-stimulating 60 times on average per session at a ~ 12:1 ratio for laser-spout vs non-laser spout. Mice receiving either D1 or D2 depolarization self-stimulated respectively at rates 7700% and 850% higher than inactive-virus EFYP control mice. Insert **(Top Left)** shows NAc placements for D1 ChR2 and D2 ChR2 mice. **(C)** Logarithmic totals. Log y-axis reveals similar trends on self-stimulation between D1 ChR2 and D2 ChR2 mice, in contrast to EYFP control mice. Bars represent log10 of average contacts across 9 days on the laser spout (red/blue/yellow) and the non-laser spout (open bars) for each group. Each dot represents an individual mouse (Males = symbols marked with a dot; Females = symbols marked with an x) and connects responses between laser-spout and non-laser spout. **(D)** Extinction (no-laser) test on Day 10 (compared to preceding Day 9 with laser). Animals received a 10^th^ session, where no laser was earned by either spout contact, and only auditory cues were delivered (laser extinction/removed) (D1 = 9 mice; D2 = 9 mice; EYFP = 12 mice). Both D1 and D2 mice virtually ceased responding when laser stimulation of ChR2 was discontinued. Inset top right shows responding for (formerly) laser and non-laser spout under extinction conditions on Day 10. Data shown are mean ±SEM; * p<0.05.

**Fig 2:**
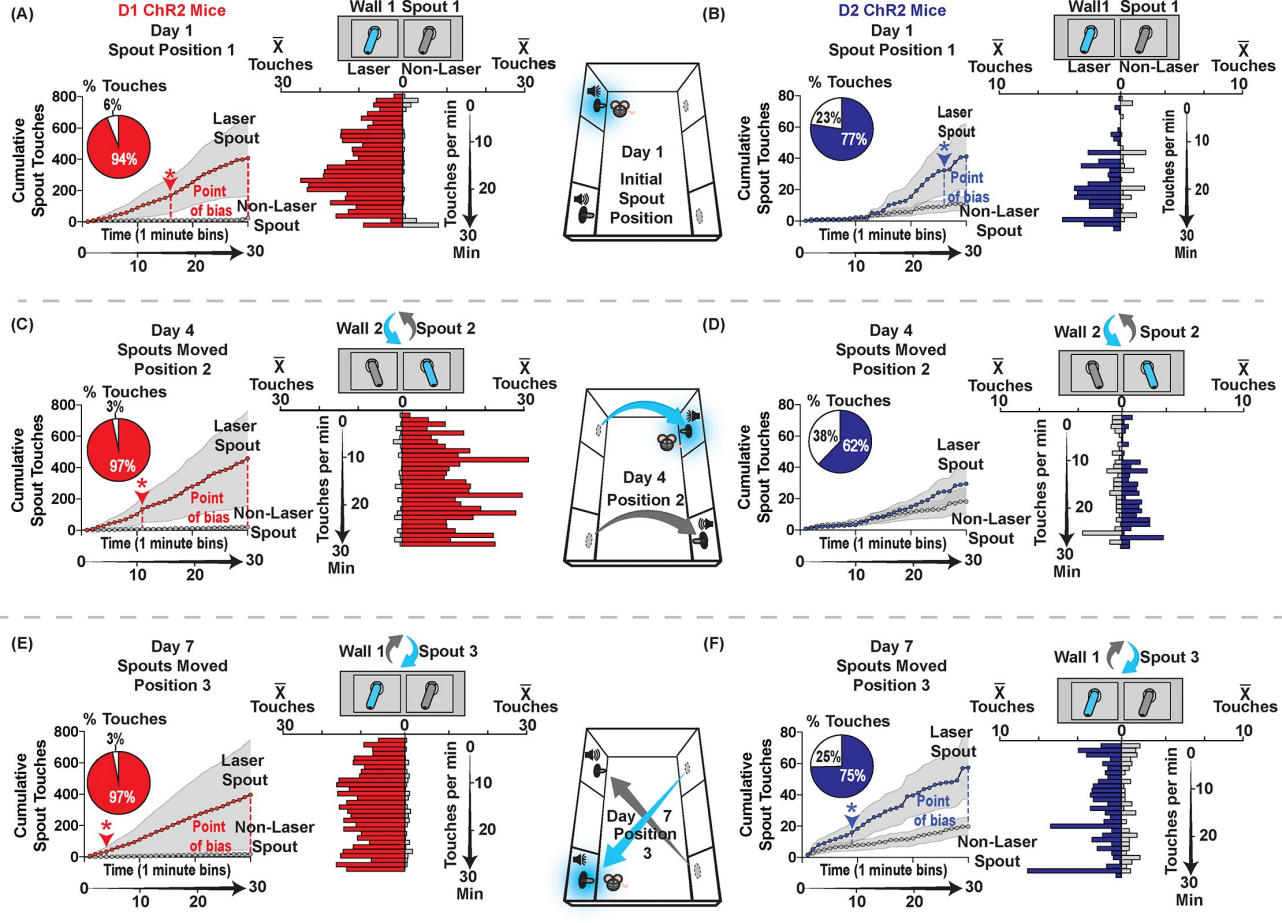
Tracking new positions: Minute-by-minute spout contacts by ChR2 mice on Day 1, Day 4 and Day 7. Responding for laser stimulation of NAc MSNs for D1 ChR2 mice (left side; n = 9) and D2 ChR2 mice (right side; n = 9) for Day 1 (**A-B**), Day 4 (**C-D**), and Day 7 (**E-F**) is shown as minute-by-minute touches (descending horizontal bars) and cumulative responding (line graph). Laser location (Position 1, 2, 3) for each day is shown in center diagram for each day and above each minute-by-minute bar graph. Touches per minute are shown by descending horizontal bars for each day. Bars projecting towards the laser spout from vertical axis show laser-spout contacts per min (red for D1 mice; blue for D2 mice). Bars projecting towards the non-laser spout from vertical axis show non-laser spout contacts for same min (gray for all mice). Cumulative touches within the day are shown by 2-line graphs at left of bar graph for each day. Point of bias indicates the minute at which cumulative contacts on laser-delivering spout becomes significantly greater than on non-laser spout. Pie charts show the percentage of laser spout contacts vs non-laser spout contacts per day. On Day 1, the position of laser-spout and non-laser spout are new, and D1 mice **(A)** begin to self-stimulate NAc ChR2 within first two minutes, while D2 mice **(B)** take about 10 minutes to begin. On Day 4, with new positions on opposite wall, D1 mice **(C)** again begin within two minutes, while D2 mice **(D)** take about 10 min to begin. On Day 7, with a third new position for each spout (reversed from Day 1), both D1 **(E)** and D2 **(F)** mice begin to self-stimulate within first minute. Data shown are mean ± SEM; * p<0.05.

Overall across 9 days of testing, D1 ChR2 mice strongly self-stimulated by specifically touching their laser-paired spout, achieving >500 laser-spout touches per 30 min session on average (SEM = +/- 103; median 58), compared to only 18 touches (SEM± 2.5; median 13) on the non-laser spout (D1 ChR2 laser vs non-laser: Wilcoxon, Z = 6.541, p < .001, r = .72; Figs 1 & 2; see S1 File for video). By contrast, D1 EYFP-control mice, infected with optically inactive-virus that lacked ChR2 in NAc, touched fewer than 10 times (SEM = +/-2.0; median 5) on either the laser spout or the alternative non-laser spout, with no difference between the two spouts, and at only 1/50^th^ of the total level of D1 ChR2 mice (EYFP laser vs non-laser: Wilcoxon, Z = 1.023, p = .306, r = .03; D1 ChR2 vs EYFP laser preference: Mann-Whitney U, Z = 7.437, p < .001, r = .54; Fig 1B). Lack of self-stimulation by inactive-virus controls confirms that mice were not simply self-stimulating for visual light or heat of intracranial laser, or delivery of an auditory cue, but rather were specifically activating neuronal D1 ChR2 photoreceptors to support D1 ChR2 levels of self-stimulation in NAc.

NAc D1 self-stimulation was rapidly acquired within a few minutes on the first day of the spout task, reaching statistical significance by the 16^th^ minute (Laser vs non-laser 16^th^ -30^th^ min; Friedman’s, *X*^2^ = 4.654, p = .031; Fig 2A). In total on Day 1, D1 ChR2 mice reached >400 contacts (SEM ±241.8; median 55) on their laser-spout, but only 26 touches (SEM± 13; median 13) on the control spout (D1 ChR2 Day 1 laser vs non-laser: Wilcoxon, Z = 2.492, p = .013, r = .83).

On subsequent Days 2 and 3 (with spouts kept in same positions as Day 1), D1 mice continued to self-stimulate NAc at high levels: always >400 stimulations (SEM± 254; median 78 and 72 for Day 2 and 3) for every mouse per 30-min session, and over 1,000 laser-spout touches for a few individuals (Day 2: Wilcoxon, Z = 2.490, p = .013, r = 83; Day 3: Wilcoxon, Z = 1.960, p = 0.05, r = .65). By comparison, EYFP mice touched the laser spout fewer than 6 times per session (SEM±1.1; median 3.5 and 5.5), and much less than D1 ChR2 mice each on Day 2 (Wilcoxon, Z = 2.110, p = .034, r = .46) and on Day 3 (Day 3 Wilcoxon, Z = 2.635, p = .007, r = .57).

On Day 4 the locations of laser-delivering spout and non-laser spout were both moved to the opposite wall. D1 ChR2 mice immediately moved to their new laser spout location and began self-stimulating within the first minute of Day 4, reaching statistical preference by the 11^th^ minute (Friedman’s, X^2^ = 5.188, p = .023; Fig 2C), and >500 contacts (SEM±301.5; median 73) on the laser spout for the entire session vs <25 contacts (SEM±13; median 10) on the non-laser spout (Entire 30 minute session, laser vs non-laser: Wilcoxon, Z = 1.836, p = .066, r = 0.612; Fig 2C). By comparison, control EYFP mice failed to touch either spout more than 13 times (SEM±3.6; median 7) on Day 4, and remained far lower than D1 ChR2 mice (Mann-Whitney U, Z = 2.171, p = 0.030, r = 0.47). On days 5 and 6, D1 ChR2 mice continued to self-stimulate NAc at levels >400 laser pulses per session (SEM±289; median 46 and 60), far above EYFP mice that touched either spout equally, and only <20 times (SEM±11.1; median 7.5 and 4.0; EYFP vs D1 ChR2 Day 5: Z = 2.101, p = .036, r = 0.45; Day 6: Z = 2.813, p = .005, r = 0.61).

On Day 7 the locations of the laser spout and non-laser spout were again switched to the original wall, but now in reverse positions from Days 1–3, so that the laser-delivering spout now occupied the initial location of the non-laser spout, and vice versa. D1 ChR2 mice again followed their laser-delivering spout to its new location nearly within the first minute on Day 7, reaching by the 3^rd^ minute significantly more touches on the laser-spout than the non-laser spout (Friedman, X^2^ = 4.571, p = .033; Fig 2E), and nearly 400 self-stimulations (SEM± 351; median 45) for the entire session (versus 13 touches on their now-inactive spout (SEM± 4.1); a >30:1 ratio; Wilcoxon, Z = 2.192, p = .028, r = 0.73). By contrast, EYFP control mice again hardly touched either spout, each < 10 times (SEM ±3.3; median 2) in the session (Mann-Whitney U, Z = 2.398, p = .015, r = 0.52). Similarly, on subsequent Days 8 and 9, with spout positions the same as on Day 7, D1 ChR2 mice continued to self-stimulate NAc at several hundred laser pulses per session (Day 8: Wilcoxon, Z = 1.482, p = .138, r = 0.49; Day 9: Wilcoxon, Z = 2.31, p = .021, r = 0.77). By contrast, EYFP control mice touched fewer than 25 times on the laser spout (SEM 7.4±; median 4.5) on Days 8 and 9, remaining far below D1 ChR2 mice (Day 8: Mann-Whitney U, Z = 2.293, p = .023, r = 0.50; Day 9: Mann Whitney U, Z = 3.490, p = .0005, r = 0.76).

Across all 9 test days, a slight trend was noted for EYFP control D1 mice to mildly prefer their laser spout over the alternative spout by nearly 2:1, but this failed to reach significance on any day (9 touches SEM±4 on laser spout, 4 SEM±3 on non-laser spout). D2-EYFP controls also showed a slight 3:2 bias toward laser spout, though also non-significant. Overall, it is clear that laser heat or visual light alone could not have motivated the high rates of NAc self-stimulation for either D1 ChR2 mice or D2 ChR2 mice here, even though rodents are reported to sometimes work for mere visual light stimulation [41].

### D2-Cre patterns of spout self-stimulation

Overall, D2 ChR2 mice also positively self-stimulated on the spout-touch task, though at relatively modest rates of about 60 laser illuminations per session (SEM± 9.6; median 29), yet still significantly above EYFP control rates of roughly 10 (SEM±1.4; median 6) illuminations (Mann-Whitney U, Z = 6.88, p < .001, r = 0.50; Fig 2; S1 File). Nearly all D2 ChR2 mice touched their laser-delivering spout at least 400% more often than the non-laser spout (Wilcoxon, Z = 6.193, p < .001, r = 0.69).

D2 ChR2 self-stimulation was relatively slower to emerge on Day 1 than in D1 mice: D2 ChR2 mice had made only 5 laser-spout touches by the 15^th^ minute (a level the D1 ChR2 mice had reached within their first 5 min; Fig 2B), and took 25 min to become statistically elevated over the non-laser spout (25^th^ -30^th^ minute laser vs non-laser spout cumulative contacts: Friedman’s X^2^ = 4.596, p = .032; Day 1 D2 ChR2 laser vs non-laser: Wilcoxon, Z = 2.10, p = .036, r = 0.70; D2 ChR2 vs EYFP: Mann-Whitney U, Z = 1.996, p = .046, r = 0.44). However, once achieved, the D2 ChR2 self-stimulation rate remained stable at 40–70 NAc (SEM±25.1; median 17 and 48) laser-spout contacts per session on subsequent Days 2 and 3, while inactive spout contacts remained < 10 (SEM±2.58; median 6) per session (6:1 ratio; Day 2 laser vs non-laser: Wilcoxon, Z = 2.524, p = .012, r = 0.84; Day 3: Wilcoxon, Z = 2.073, p = .038, r = 0.69; Day 2 D2 ChR2 vs EYFP: Mann-Whitney U, Z = 2.147, p = .032, r = 0.47; Day 3 D2 ChR2 vs EYFP: Mann-Whitney U, Z = 2.068, p = .039, r = 0.45).

### D2 ChR2 mice slowly track spout shifts in location

On Day 4, when both spouts were shifted to the opposite wall, D2 ChR2 mice initially failed to track the active laser spout to its new location on that day, making ~30 (SEM±10.5; median 19) or so laser spout contacts vs 20 control-spout contacts (SEM± 8.0;D2 ChR2 laser vs non-laser: Wilcoxon, Z = 1.820, p = .069, r = 0.61; D2 ChR2 vs EYFP: Mann-Whitney U, Z = 1.71, p = 0.87, r = 0.37; Fig 2D). However, they began to track on Day 5, and by Day 6 were again self-stimulating at about 70 NAc laser-spout (SEM± 25.3; median 55) contacts vs 18 (SEM±4; median 16) on control-spouts, a > 3:1 ratio (Wilcoxon, Z = 2.31, p = .021, r = 0.77), while EYFP-controls remained at 7–15 contacts (SEM±3.27; median 4) at both spouts (D2 ChR2 vs EYFP, Day 6; Mann-Whitney U, Z = 2.563, p = .010, r = 0.56).

On Day 7 the laser spout was moved to a third location (original wall, but laser and non-laser spout were reversed in position from their Day 1–3 locations). This time, D2 ChR2 mice did successfully track the laser spout to its new location on the same day, reaching significant self-stimulation levels by the 8^th^ minute of Day 7 (Fig 2E; Cumulative minute by minute laser v non-laser: Friedman’s X^2^ = 4.587, p = .032), and earning nearly 60 NAc self-stimulations (SEM± 20.2; median 32) in the session, compared to only 20 touches (SEM±5.7; median 13) on the non-laser spout (Day 7 D2 ChR2 laser vs non-laser: Wilcoxon, Z = 2.312, p = 0.021, r = 0.77). On subsequent Days 8 and 9, D2 ChR2 mice continued to self-stimulate at levels of >60 (SEM±24.2; median 41) and >120 (SEM±57.4; median 74) illuminations per session, respectively (Day 8: Wilcoxon, Z = 2.134, p = 0.033, r = 0.711; Day 9: Wilcoxon, Z = 2.380, p = 0.017, r = 0.79). By contrast, EYFP control mice showed no preference for either spout and remained low on both spouts on all days (12 touches SEM± 3 on laser-spout to 11 SEM±2 on alternative spout; D2 ChR2 vs. EYFP Day 7–9: Kruskal-Wallis, X^2^ = 18.91, p < .001).

### Self-stimulation immediately declines during laser extinction

On Day 10, the laser was discontinued for all groups, and only the Pavlovian auditory cues (conditioned stimuli or CSs) were earned by spout touch (Fig 1D). In laser extinction, D1 ChR2 mice quickly declined within a few minutes of the laser-extinction session, and in total made only 10% of their previous day’s level of touches on the former laser-spout in the 30-min session (460 touches SEM±240 vs 48 SEM±15.6; Wilcoxon, Z = 2.201, p = .028, r = 0.59). Similarly, D2 ChR2 mice also immediately declined within minutes to <15–20% of their previous day’s level of touches on the formerly-active spout, and no longer differed from their EYFP control counterparts in total contacts (132 touches SEM±57.4 vs 18.6 SEM±7.5; D2 ChR2 vs EYFP Extinction: Mann-Whitney U, Z = .20, p = .328, r = 0.26). Thus, it appeared that laser stimulation of ChR2 in NAc was required to maintain expression of self-stimulation levels, both for D1 and D2 mice. Neither a stimulus-response habit nor conditioned reinforcement by associated CSs were sufficient to maintain previously-established levels of spout contacts in the absence of actual laser stimulation.

### D1 vs D2 comparison for NAc self-stimulation on 9-day spout-touch task

Overall, contrasting D1 mice to D2 mice, D1 ChR2 self-stimulation levels were nearly an order of magnitude higher than D2 ChR2 levels (>800%; Mann-Whitney U, Z = 2.627, p = .009, r = 0.21; Fig 1B). D1 ChR2 levels reliably achieved >500 NAc self-stimulations (SEM±103; median 148) per daily session, and a few D1 ChR2 individuals exceeded 2000 self-stimulations per session. By comparison, D2 ChR2 levels remained at about 60 self-stimulations (SEM±9.6; median 38) per session on average. The top few D2 individuals reached a maximum of only 100 to 200 per session on average, with one mouse reaching a one-time maximum of ~500 contacts. For the non-laser spout, both D1 ChR2 and D2 ChR2 mice touched at equivalent rates of about 15–20 times per session (Mann-Whitney U, Z = 0.505, p = .614, r = 0.04). This divergent pattern for the laser spout suggests that D1 levels of NAc ChR2 self-stimulation were reliably much higher than D2 levels. Further, similar rates of touching the non-laser spout implies the difference in self-stimulation was clearly not due to simple differences in general activity or spout interest between D1-Cre mice and D2-Cre mice, but rather reflected true differences in appetitive motivation for selective NAc excitation.

### Passive location-based self-stimulation: Only D1 ChR2 mice seek out laser location

In the separate location-based self-stimulation task, simply entering or moving while within the designated laser-corner triggered an infrared motion detector. Every activation of the laser-paired motion detector delivered a 1-sec constant-illumination 1 mW laser pulse to NAc. The square chamber contained an occluding cylinder in the center, allowing mice to explore only the periphery and all four corners as they chose, but not to enter the center of the square. Only one corner delivered laser on any given day. In this chamber, mice typically ran almost continuously around the periphery, usually in the same direction throughout the session as if on a running track (usually in counter-clockwise direction). Running was interrupted by occasional brief 1–3 sec pauses in corners. The laser-designated corner remained constant throughout the entire first session, but then was switched to a different corner the next day and switched again to a third new corner on the 3^rd^ day.

### Overall location stimulation preference

Initial mixed between/within ANOVAs revealed that corner preferences differed both between D1 and D2 ChR2 mice, and also varied by particular test day (Two-way ANOVA: Day x D1 vs D2: *F*_(2, 54)_ = 3.511, *p* = .037; Day: *F*_(2, 54)_ = 3.511, *p* = .059; D1 vs D2: *F*_(1,27)_ = 1.413, *p* = .245). Follow up analyses were performed using non-parametric statistics, as data did not conform to parametric standards (D1 ChR2 laser-paired corner: Shapiro-Wilk, W = .918, df = 26, p = .04; D2 ChR2 laser-paired corner: W = .904, df = 27, p = .016).

### D1 mice: Positive preference for NAc ChR2 laser corner

**Fig 3:**
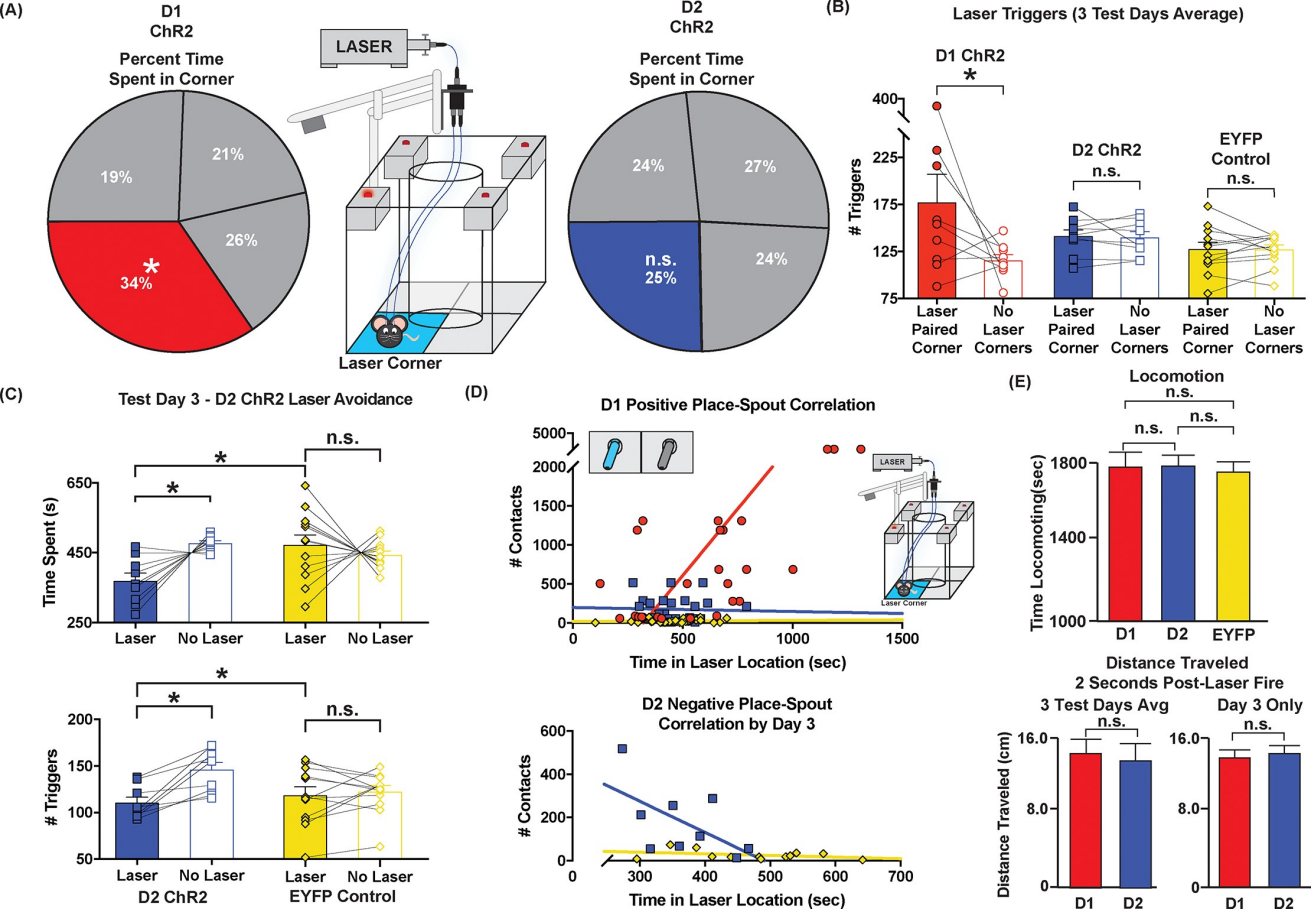
Location based NAc ChR2 task: D1 self-stimulation by corner preference, D2 failure to prefer. A square chamber, with center occluded, allowed mice to earn NAc laser stimulations by entering each day’s laser-designated corner. An infrared motion detector in that corner triggered a 1mW, 1s, constant pulse of laser upon entry and again upon each subsequent detected movement (30-min session). The laser-designated corner moved each day for three days. **(A) Time spent:** D1 mice (n = 9) significantly spent more time in their laser corner each day than in any other corner. By contrast, D2 ChR2 (n = 9) mice showed no preference for any corner overall. **(B) Number of corner detector triggers:** D1 mice earned an average of approximately 180 ChR2 laser bins via their laser-corner, whereas D2 mice and inactive-virus control mice received only two-thirds of that amount, respectively. Non-laser corner shows the average earned across the 3 non-laser corners combined **(C) D2 ChR2 mice laser avoidance**: By Day 3, a majority of D2 ChR2 mice (n = 7/9) mostly showed avoidance of laser-paired locations reducing the number of laser triggers, and spending only 19% of their time in the laser corner. **(D) Correlation between spout-touch vs place-based self-administration:** D1 ChR2 spout self-stimulation is correlated with stronger preference for laser-paired locations. By contrast, D2 ChR2 mice show no correlation between corner preference and spout touches. In fact, D2 individuals with highest self-administration in spout-task were most likely to avoid laser-corner in the location-based task by Day 3. **(E) Locomotion: (top)** D1, D2, and EYFP control mice all showed similar amounts of time in locomotion overall during the session. Similarly **(bottom)**, D1, D2 and EYFP mice did not differ in locomotion distance traveled during actual illuminations (i.e., during 2-sec periods that began with laser initiation), indicating that differences in corner preference were not simply due to D1 vs D2 differences in motor effects. Data shown are mean ±SEM; * p<0.05.

Across the 3 days of testing, D1 ChR2 mice continually preferred their NAc laser-delivering corner and followed it to new locations as it moved: reliably pausing and spending about 150% more time in that corner than in any other corner (Friedman One Way, X^2^ = 9.643, p = .022; Fig 3). Overall, D1 ChR2 mice triggered about 180 cumulative NAc laser stimulations per session (SEM±19.4; median 153) and reached that level of preference as early as Day 1. By comparison EYFP virus control mice showed no preference, essentially distributing their time equally across all four corners, and overall received significantly fewer laser illuminations at only about two-thirds of D1 ChR2 levels (120 sec SEM±6.9; median 100; Kruskal-Wallis, X^2^ = 5.549, p = .018).

In general, comparing individual D1 ChR2 performance on place-based versus spout-based self-administration tasks, there was a significant positive individual correlation between self-stimulation on the two separate tasks: D1 individuals with highest numbers of total spout touches tended to also spend most time in their laser corner (Fig 3D; Spearman’s rho = 0.649, p<0.001).

### D2 NAc ChR2 mice fail to prefer laser corner

D2 ChR2 mice as a group did not detectably prefer the NAc laser-delivering corner on any day, nor overall (Days 1–3 time spent: Friedman’s, X^2^ = 4.911, p = .178; Fig 3). Instead on the first and second day, all D2 ChR2 mice spent equal time in all four corners, and did not differ in duration, entries or movements across corners, apparently ignoring laser location (Day 1 Time: Friedman, X^2^ = 4.333, p = .228; Day 1 motion detector triggers: Kruskal-Wallis, X^2^ = .4.067, p = .254; Day 2: Time Spent: Kruskal-Wallis, X^2^ = 0.733, p = .865; Day 2 motion detector triggers: X^2^ = 3.267, p = .352).

However, on Day 3 individual differences appeared to emerge within D2 ChR2 mice. In particular, 7 out of 9 D2 ChR2 individuals avoided the laser corner compared to all other corners, even when the laser corner moved to a new location (All D2 ChR2: Friedman’s, X^2^ = 9.944, p = .019; Fig 3C). One of these laser-avoiding D2 mice reliably paused prior to entry to its laser corner, while the other 6 D2 ChR2 mice in this group entered the corner, but then escaped the laser corner more rapidly than after entry to other corners (i.e., within 1–2 seconds of laser onset). This laser avoiding/escaping group of D2 ChR2 mice also spent less time in the laser corner than EYFP control mice did on Day 3 (Kruskal-Wallis, X^2^ = 4.854, p = 0.028), as EYFP mice continued to ignore laser location and spent equal time in all four corners (EYFP Time Spent: Friedman’s, X^2^ = 6.051, p = .109).

In passing, we noted that D2 mice which avoided/escaped their laser corner on Day 3 had previously shown reliable positive self-stimulation on the earlier spout task. We therefore returned these mice to the spout-task for retesting to assess if they would still self-stimulate by spout touches. Spout results showed that these D2 mice still positively self-stimulated by making several dozen to >100 contacts on their laser spout, ~10 on the non-laser spout. Thus, unlike for D1 mice, for D2 mice there was no correlation between laser-corner preference in the place-based task and spout-touch self-stimulation in the spout task (Fig 3D; Spearman’s rho = -0.048, p = 0.812). The two measures of self-stimulation were independent and different even for the same individuals.

In terms of general locomotion, all D1 ChR2 and D2 ChR2 mice showed comparably high levels of running, spending nearly 90% of time in the chamber in motion (D1 vs D2 ChR2 Time Locomotion; Kruskal Wallis, X^2^ = .308, p = .579). Similarly, EYFP D1 and EYFP D2 control mice were comparable in locomotion to ChR2 mice (D1 vs EYFP Time Locomotion: X^2^ = 426, p = .670; D2 vs EYFP Time Locomotion: Kruskal Wallis, X^2^ = 2.515, p = .113). Further, D1 ChR2 and D2 ChR2 mice traveled similar distances during actual laser illuminations (2-second periods following laser onset) across all 3 test days (D1 ChR2 Distance = 15.43cm SEM+/- 1.92 vs D2 ChR2 Distance = 13.93cm SEM+/- 1.80; Two-Way ANOVA: D1 vs D2 ChR2 X Test Day: *F*_(2, 26)_ = .241, *p* = .787; Main Effect of D1 vs D2 Condition: *F*_(2, 32)_ = .324 *p* = .579; Main Effect of Test Day: *F*_(2, 32)_ = .241 *p* = .787). Additionally, no differences were observed between D1 and D2 ChR2 mice in the distance traveled following laser pulses on Test Day 3(D1 = ~13.5cm (SEM+/- .044) and D2 = ~14 cm (SEM+/- 3.03), respectively (Mann-Whitney U, *Z* = 0.210, p = .834).Therefore, overall, we found no D1/D2 differences in locomotion overall, nor specifically induced by NAc laser, in this 4-corner arena.

### NAc sites and localization of function analyses

#### NAc self-stimulation sites

**Fig 4:**
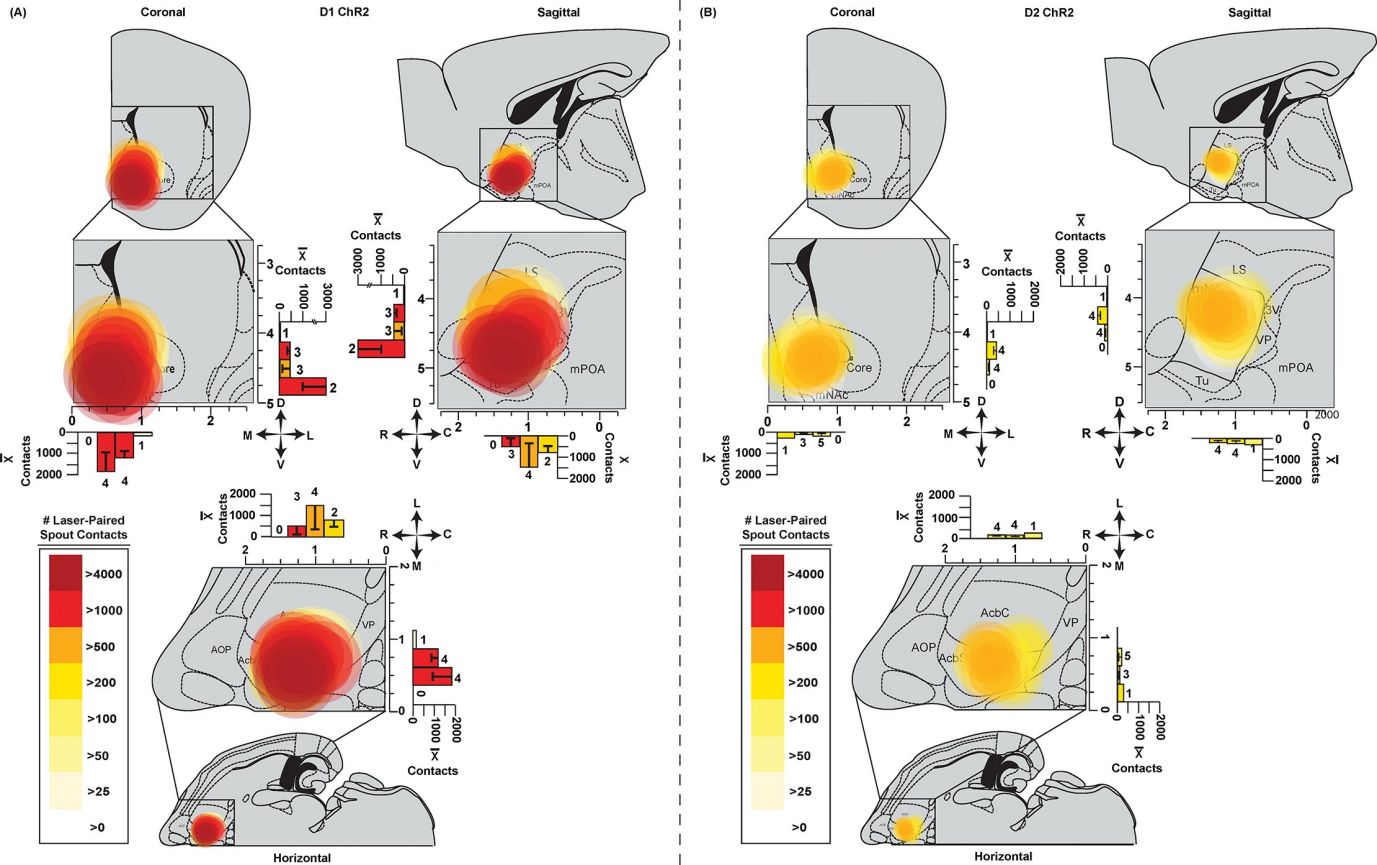
Anatomical sites in NAc: Shell and core sites support self-stimulation. Circle symbol locations show individual D1 **(A)** or D2 **(B)** sites in coronal, sagittal, and horizontal planes. Circle colors show the level of self-stimulation supported at each site (measured in the same mouse). Symbol sizes show the mean diameters of concentric Fos plumes (produced by laser illumination of ChR2 prior to perfusion). Two fiber optic placements per animal for D1 mice (n = 9) or D2 (n = 9) mice are mapped on to coronal, sagittal, or horizontal images. **(A)** D1 sites were within NAc medial shell or core which supported similar levels of 500 to 4000 self-stimulations per spout session. **(B)** D2 ChR2 sites were similarly within either NAc shell or NAc core, and generally supported self-stimulation at levels between showed moderate rates of 50 to 400 self-stimulations per session.

In D1 ChR2 mice, NAc sites for optic fiber tips were clustered mostly in the medial shell of NAc (n = 5), filling most of its anterior-posterior and dorsal-ventral dimensions as a group, or else in medial NAc core (n = 3) or on the border between shell and core (n = 1) (Fig 4A). Anatomical sites were mapped for self-stimulation efficacy to detect any potential localizations of function (Fig 4). However, all sites in NAc appeared to support comparable levels of D1 self-stimulation, whether in medial shell or core, and we did not detect statistical localization of function differences within NAc between either core or shell, or between A-P or D-V zones of shell (medial shell = ~1600± 802 laser spout touches; one high individual D1 ChR2 >4500; core = ~274±242 touches overall highest individual ~700).

**Fig 5:**
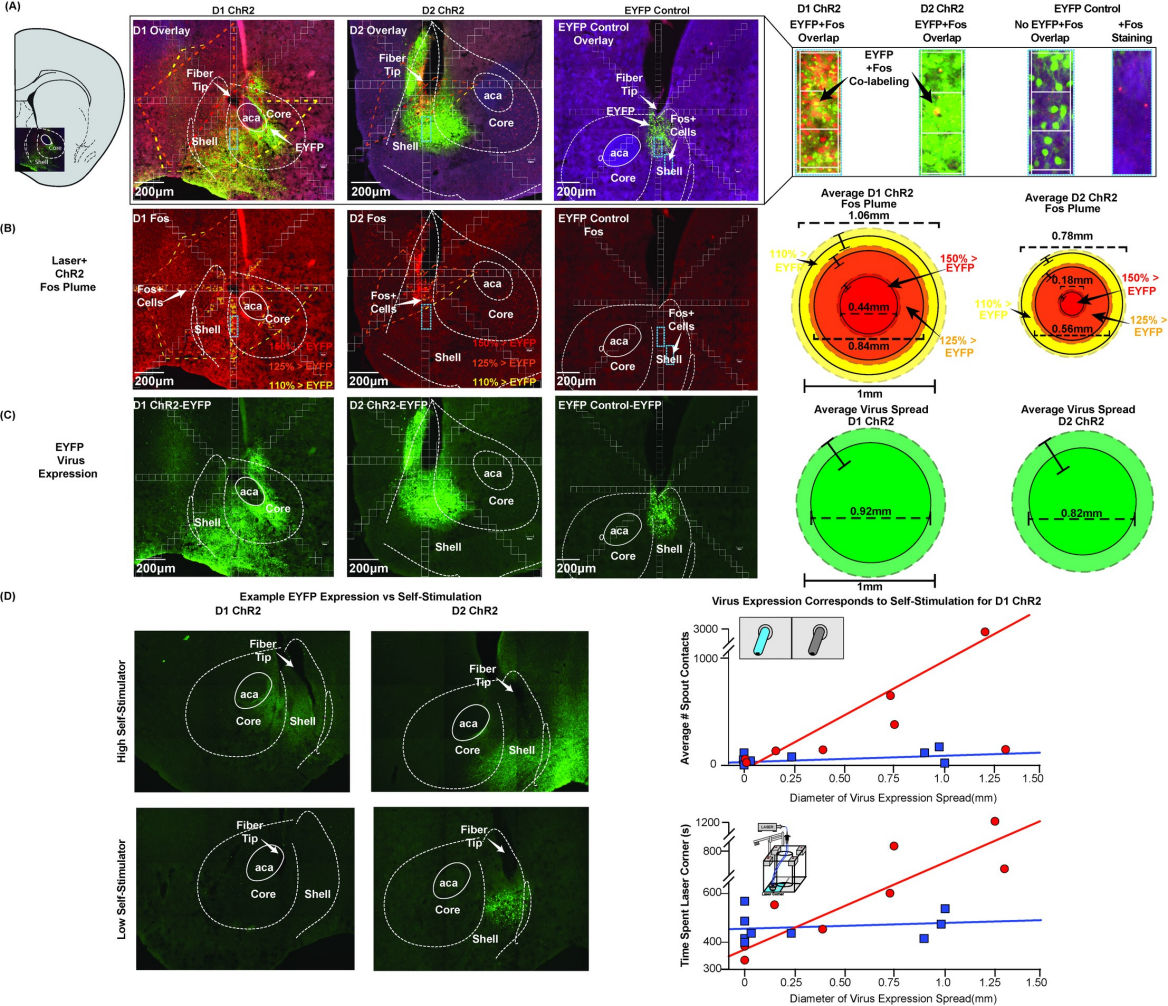
Laser-induced Fos plumes and virus expression in NAc. **Top (A):** Overlay of immunohistochemistry-labeled Fos protein expression (AlexaFluor488; red), virus expression (enhanced yellow florescent protein; EYFP; green), and calculated Fos plume boundaries (% Fos elevation induced by laser + ChR2 over EYFP baselines). Blue outlined insets show close-up 50um square boxes used to count neurons with Fos expression, and to assess viral spread within radial arms. ChR2 or EYFP-control mice here all received laser stimulations immediately before euthanasia. **(B)** Fos examples from D1 mice are in left column; D2 mice are in the middle; EYFP control mice are in right column after receiving laser stimulation. Squares (50μm x 50μm) emanate along radial arms from the center of the fiber tip. Individual plume boundaries assessed by different intensities of laser+ChR2 Fos elevation above EYFP control baselines (>110%; >125%; >150% indicated by colors of dashed lines). Mean diameters of Fos plume intensity zones are shown right. D1 ChR2 stimulation produced a 0.44mm (SEM± 0.050) inner plume reaching 150% elevation of Fos above EYFP control, with a larger 0.84mm (SEM± 0.065) diameter middle plume of >120% elevation of Fos, surrounded by an even larger 1.06mm (SEM± 0.037) diameter outer plume of >110% elevation. D2 ChR2 plumes had lower intensity centers of >150% Fos elevation with 0.18mm diameter (SEM± 0.029), a 0.58mm (SEM± 0.049) diameter middle plume of >120% enhancement, and an outer 0.78mm plume (SEM± 0.036) of >110% Fos enhancement. **(C)** Virus spread alone: D1 mean diameter of 0.92mm (SEM± 026mm), and D2 diameter of 0.82mm (SEM±0. 22mm). **(D) (Left)** Example images of the highest self- and lowest stimulating D1 or D2 ChR2 mice. **(Right)** Virus expression correlates with D1 ChR2 mouse self-stimulation on both spout-tasks and location-based stimulation, but not for D2 ChR2.

D2 ChR2 sites were similarly clustered in NAc medial shell (n = 6), medial core (n = 2), or on the core-shell border (n = 1) (Fig 4). Assessment of localization of function again indicated that D2 ChR2 shell and core sites had comparable levels of self-stimulation (D2 ChR2 shell = 165±76 for group, with highest individual at >500; core = 271±97 and highest individual at 250; Fig 5B). Again, we did not detect any systematic anatomical differences in D2 ChR2 self-stimulation rates across NAc sites in core or shell.

#### Virus infections

D1 ChR2 mice had virus infection diameters in NAc of about 0.92 mm (SEM+/- 0.22mm) assessed by fluorescent protein expression, and D2 ChR2 mice had similar virus diameters of 0.82mm (SEM+/- 0.26mm) (D1 ChR2 vs D2 ChR2 Viral Spread Kruskal Wallis, *X*^2^ = 0.145, p = .703). However, a few D1 ChR2 and D2 ChR2 individuals had larger virus infections, reaching nearly 3mm in diameter. Virus infections thus typically filled the entire nucleus accumbens shell and core, and in the larger cases often extended into other structures such as lateral septum, olfactory tubercle, and rarely ventral portions of dorsal striatum.

The diameters of D1 ChR2 viral infections in NAc was positively correlated with the behavioral intensity of spout self-stimulation (D1 ChR2 Spout-Self Stimulation vs Virus Spread Distance: Spearman’s rho = .731, p = .040). D1 ChR2 infection diameter was also positively correlated with the behavioral strength of laser-corner preference in the place-based self-administration task (Time spent in laser location vs virus spread distance: Spearman’s rho = 0.898, p = 0.002). However, despite those correlations, the highest self-stimulating D1 ChR2 mouse, achieving on average ~2800 spout contacts per 30-min session, did not have the widest viral spread. Conversely, the D1 ChR2 mouse largest viral spread of 1.3mm in diameter averaged only ~150 spout contacts per session.

For D2 ChR2 mice, the diameter of viral infection did not reach significance in correlation with either spout self-stimulation (D2 ChR2 spout-self stimulation vs virus spread distance: Spearman’s rho = .174, p = .654) or place-based laser corner preference (Time spent in laser location vs virus spread distance: Spearman’s rho = 0.218, p = .574). The highest D2 ChR2 self-stimulator earned approximately 160 laser pulses per session and had a virus diameter of 0.98mm, whereas one of the lowest D2 ChR2 mice earned only ~14 laser pulses per session and had comparable virus spread of 1mm diameter. Additionally, at roughly equal diameters of virus expression, D1 ChR2 mice still tended to show higher levels of self-stimulation behavior than D2 ChR2 mice.

#### Sizes of local Fos plumes in NAc

Laser illumination in D1 ChR2 mice and D2 ChR2 mice produced local Fos plumes of elevated Fos expression immediately surrounding the optic fiber tips, producing the greatest Fos enhancement immediately beneath the fiber optic tip. Local Fos plumes are presumed to reflect the diameter and intensity of local neuronal stimulation induced by illumination of surrounding ChR2-infected neurons [42]. The diameters of Fos plumes induced by 1 mW ChR2 illuminations were approximately 0.3mm (intense >150% Fos elevation center) to 1.0 mm in outer diameter (moderate >110% Fos elevation surround) in both D1 and D2 mice (laser stimulation was given immediately prior to perfusion; Fig 5). Thus, Fos plumes were much smaller than zones of virus infection. The outer diameters of Fos plumes were only one-half the diameters of virus infections, and the inner plumes zones of more intense Fos elevations were even smaller relative to virus infections. This difference suggests that laser illumination altered only a portion of virus-infected neurons, namely those within 0.5 mm radius of the optic fiber tip, and most intensely neurons within 0.02 to 0.2 mm of tip as described below. This suggests that only a small portion of infected neurons receive sufficient illumination to alter Fos production, and presumably neuronal function. If so, Fos plumes within a virus cloud provide a more accurate estimate of the zone of neuronal activation than the virus infection *per se*.

In D1 mice, ChR2 Fos plumes contained an inner 0.44mm diameter (SEM ±0.050) center of intense >150% Fos elevation (i.e., 1.5 times above the 100% control levels of EYFP D1 mice that also received laser illumination), surrounded by a larger 0.84mm diameter (SEM ±0.065) middle plume of more moderate >120% Fos elevation, and finally by a still-larger 1.06mm (SEM ±0.037) outer plume of mild >110% elevation (D1 ChR2 vs EYFP Fos plume: Kruskal Wallis *X*^2^ = 13.19, p < .001). This outer plume diameter of approximately 1.06mm was used to set the largest diameter of D1 ChR2 symbols in NAc self-stimulation maps, with concentric circles showing the inner >150% and middle >125% zones (Fig 5).

In D2 mice, ChR2 Fos plumes induced by laser illumination were similar in diameter to plumes of D1 mice, but with typically less intense Fos elevation. ChR2 illuminated local Fos plumes produced by laser illumination similarly contained an outer 0.78mm-diameter (SEM ±0.036) plume of milder >110% Fos elevation. Inside was a middle 0.58 mm-diameter (SEM ±0.049) plume of moderate >125% Fos elevation, and an inner plume of only 0.18mm-diameter (SEM ±0.029), of intense >150% Fos elevation (elevations calculated from baselines in EYFP control mice that also received laser illumination before perfusion; D2 ChR2 vs EYFP Fos plume: Kruskal Wallis X^2^ = 27.94, p < .001). The, outer plume diameters of approximately 0.8mm were used to maximally size D2 map symbols, with inner symbols of 0.18mm diameters (Fig 5). Taken as groups, both the D1 ChR2 and D2 ChR2 Fos plumes filled over 90% of the entire medial shell, and at least the most medial portion of core.

#### NAc D1/D2 stimulation recruits similar distant Fos activation in other brain structures

**Fig 6:**
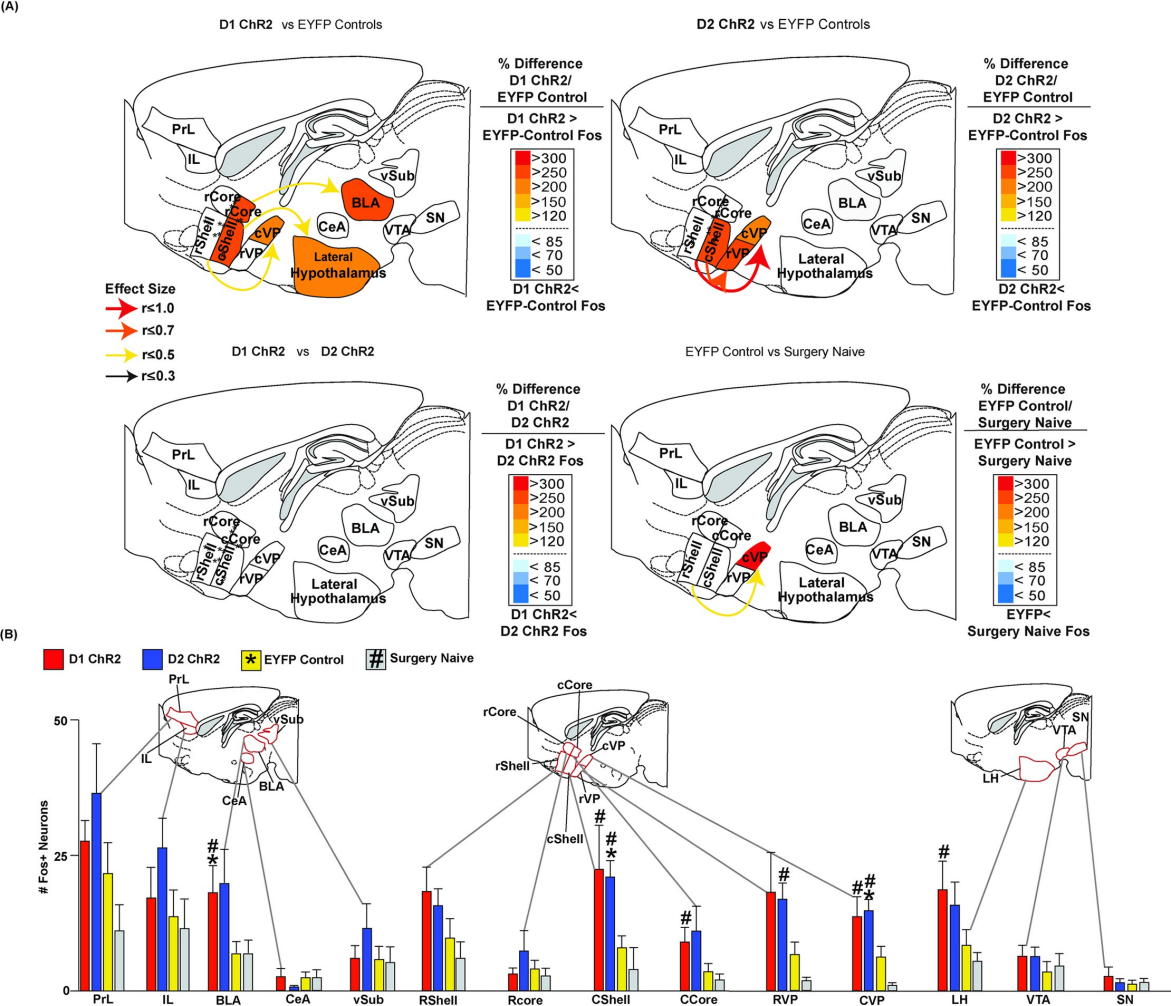
D1 vs D2 maps of distant Fos recruitment in limbic structures. **(A)** Sagittal maps show relative Fos elevations in each structure induced by NAc laser stimulation in D1 ChR2 mice compared to EYFP control mice **(top left)**, D2 ChR2 mice compared to EYFP controls **(top right)**, and a direct contrast between D1 ChR2 mice and D2 ChR2 mice (**bottom left**). A baseline comparison map **(bottom right)** shows elevation of Fos in EYFP mice over unoperated naïve D1/D2 control mice. Both D1 ChR2 and D2 ChR2 stimulations in NAc produced similar anatomical patterns of distant Fos changes in other brain structures. Consequently, and D1 vs D2 ChR2 patterns of distant Fos expression did not significantly differ for any limbic brain structure sampled here. In each map, arrow size denotes effect size of Fos change (assessed using the formula $r=\frac{Z}{\sqrt{N1+N2}}$ and color of structure denotes percentage change in Fos in that structure. **(B)** Bar histograms showing mean (+/-SEM) absolute levels of Fos expression for each group/structure. Fos-expression neurons were counted within a 600x800-micron rectangular box sample from each indicated structure or subregion, to standardize sample volume across different structures. Bilateral samples were taken per mouse (one per hemisphere) and averaged for a single Fos count. Red bars = D1 ChR2 mice; Blue = D2 ChR2 mice; Yellow = EYFP mice; gray = unoperated/surgically-naïve control mice. * = different from EYFP control at p = 0.05. # = different from surgery naïve controls at p = 0.05. Brain region abbreviations are: Prelimbic cortex (PrL), Infralimbic cortex (IL), Basolateral Amygdala (BLA), central nucleus of Amygdala (CeA), ventral subiculum (vSub), rostral NAc Shell (rShell), rostral NAc core (rCore), caudal half of NAc medial shell (cShell), caudal NAc Core (cCore), rostral ventral pallidum (rVP), caudal Ventral pallidum (cVP), lateral hypothalamus (LH), ventral tegmental area (VTA), substantia nigra (SN).

Beyond NAc, laser stimulations also recruited increases in distant Fos in several other limbic brain structures, in both D1 ChR2 mice (n = 6) and D2 ChR2 mice (n = 5) (Fig 6). Distant Fos was measured in the ventral pallidum, lateral hypothalamus, ventral tegmental area, substantia nigra, basolateral nucleus, central nucleus of amygdala, subiculum of hippocampus, prelimbic, and infralimbic cortex. To assess elevations induced by NAc photoexcitation versus optogenetic virus infection and surgery, we measured two control baseline levels in different groups: 1) inactive-virus EYFP D1/D2 mice that similarly received laser before perfusion (n = 13; 7 D1 + 6 D2), and 2) normal unoperated D1 and D2 mice that never received any surgery (n = 6).

Initial 2-way ANOVA indicated laser-induced Fos elevation differences in downstream brain regions based on ChR2 or EYFP treatments in D1 or D2 neurons (Two-Way ANOVA: brain region xChR2 vs EYFP: *F*_(39, 325)_ = 1.74, *p* = .006; Main effect of brain region: *F*_(13, 325)_ = 19.09, *p* < .001; Main effect ChR2 vs EYFP: *F*_(3, 25)_ = 4.912, *p* = 0.008). Subsequent One-Way ANOVA comparisons indicated Fos differences between ChR2 or EYFP treatments in D1 or D2 neurons in NAc, VP, and BLA, (NAc: Kruskal Wallis, X^2^ = 9.386, p = .025; VP: Kruskal Wallis, X^2^ = 13.471, p = .004; BLA: Kruskal Wallis, X^2^ = 7.846, p = .049) and showed trending differences in LH (LH: Kruskal Wallis, X^2^ = 7.173 p = .067).

**Fig 7:**
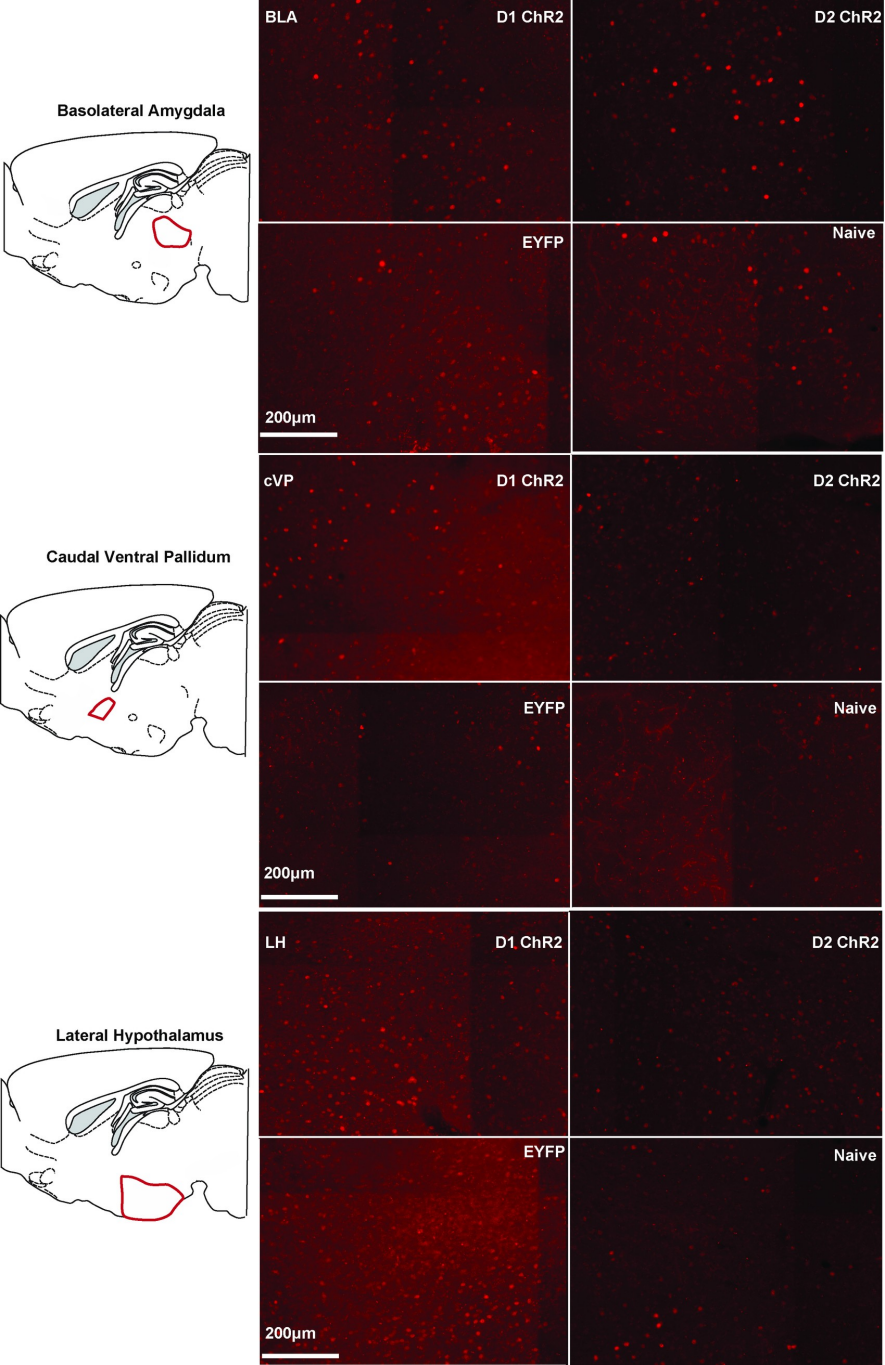
Example photomicrographs of Fos recruited in distant brain structures. Images showing Fos core samples recruited by NAc laser illumination in D1 ChR2 or D2 ChR2 mice, or controls. Distant structures shown are basolateral amygdala **(Top)**, caudal ventral pallidum **(Middle)** and lateral hypothalamus **(Bottom).** D1 ChR2, D2 ChR2, EYFP control, and surgically naïve mice are each shown. Brain region abbreviations: Basolateral Amygdala (BLA), caudal Ventral pallidum (cVP), and lateral hypothalamus (LH).

Distant elevations in cortical and subcortical structures were recruited by ChR2 NAc stimulations, and the pattern was similar in both D1 ChR2 and D2 ChR2 mice (see Fig 7 for example images). For example, in ventral pallidum, a target of NAc outputs for both D1 MSNs and D2 MSNs, illuminated D1 ChR2 mice had elevations >270% above illuminated EYFP controls, both in rostral and caudal subregions of ventral pallidum (VP: D1 ChR2 vs EYFP: Mann-Whitney U, Z = 88.00, p = .032, r = 0.492), raising them a full order of magnitude above unoperated naive mice (VP: D1 ChR2 vs naive: Mann-Whitney U, Z = 23.00, p = .002, r = 0.613). Similarly, illuminated D2 ChR2 mice were >250% above EYFP controls in ventral pallidum, both in rostral and caudal subregions (VP: D2 ChR2 vs EYFP: Mann-Whitney U, Z = 48.00, p = .004, r = 0.593), and roughly ten times the level in unoperated naïve control D2 mice (VP: D2 ChR2 vs naive: Mann-Whitney U, Z = 15.36, p < .001, r = 0.80). This suggests that mere surgery/virus /heat in NAc of EYFP controls may elevate VP Fos considerably above baseline unoperated levels, and that adding light-induced ChR2 photoexcitation of NAc neurons further elevates VP Fos levels in both D1 ChR2 and D2 ChR2 mice.

In lateral hypothalamus (Fig 7), another indirect output target of NAc shared by D1 and D2 MSNs, D1 ChR2 and D2 ChR2 stimulations both recruited similar >200% Fos elevations above illuminated EYFP control levels and were nearly 300% above levels of unoperated control (LH: D1 vs naïve Mann-Whitney U, Z = 2.22, p = .026, r = 0.62; LH: D2 ChR2 vs naïve Mann-Whitney U, Z = = 1.87, p = .061, r = 0.54). In ventral tegmentum Fos had relatively low baseline levels, and optogenetic stimulation produced no statistically significant changes.

In basolateral amygdala, D1 ChR2 laser stimulation of NAc induced significant Fos elevation of >250% above similarly-illuminated EYFP control levels (BLA: Mann Whitney U, Z = 2.07, p = .039, r = 0.47), and >270% above unoperated control EYFP mice (BLA: Mann Whitney U, Z = 2.07, p = .038, r = 0.576). By comparison, D2 ChR2 stimulation induced only a marginal trend toward elevation in basolateral amygdala, but still reached levels >280% above EYFP control levels (BLA: Mann-Whitney U, Z = 1.83, p = .067, r = 0.53), and similarly above unoperated naïve levels (BLA: Mann-Whitney U, Z = 1.802, p = .072, r = 0.52).

In prefrontal cortex, marginal trends toward Fos increases of roughly 200% were noted in prelimbic and infralimbic cortex, after NAc ChR2 stimulation in both D1 and D2 mice. D2 ChR2 mice additionally had similar trends toward increase in ventral subiculum of hippocampus.

Finally, comparing just the two baseline control groups (EYFP mice versus unoperated mice), NAc-illuminated EYFP mice typically had moderately higher 150% Fos elevations above normal unoperated mice in prelimbic and infralimbic regions, in rostral and caudal NAc shell and NAc core, LH, a 300% Fos elevation in rostral VP, and much higher 600% levels in caudal VP (Caudal VP: EYFP vs unoperated controls Mann-Whitney U, Z = 2.093, p = .036, r = 0.47;Fig 6). This pattern suggests that mere laser heat/light in NAc and/or virus infection in NAc by itself may rather powerfully recruit Fos activation in some distant brain structures. However, we stress that all the D1 and D2 Fos elevations caused by ChR2 illumination described above were always elevated above the higher EYFP control baselines. The EYFP baselines were also used for ChR2 statistical comparisons, in order to conservatively identify true ChR2 optogenetic recruitment of neuronal activation in distant brain structures.

## Discussion

These results showed that optogenetic excitation of D1 neurons in NAc shell and core supported strong self-stimulation seeking behavior in both spout-touch and location-based tasks. In the spout task, D1-Cre mice made at least several hundred active touches, and sometimes over four thousand per 30 min session, on their laser-delivering spout to earn ChR2 laser stimulations in NAc (i.e., equivalent to 2.5 spout touches per sec). When the spout moved, D1 mice nearly instantly tracked it to its new location, shifting within a minute or so. Similarly, in the location-based task, D1 mice reliably spent more time in their ChR2-stimulating laser corner, pausing longer in it than in other corners to earn significant NAc self-stimulation, and successfully followed their laser-corner as it moved each day.

By comparison, D2-Cre mice in the spout-touch task displayed lower, yet still clearly positive, levels of NAc ChR2 self-stimulation behavior. D2 ChR2 mice made at least several dozen laser-spout touches per session to stimulate NAc D2 neurons, and in a few cases earned hundreds of NAc illuminations. Though much slower than D1 mice to acquire and track initially when their laser-spout moved, D2 ChR2 mice also did eventually succeed in following to its new location the first time it shifted, and on their final shift D2 mice followed adeptly within 10 min.

However, when tested in the separate location-based task, D2 ChR2 mice mostly ignored their laser-delivering corner (unlike D1 ChR2 mice) and failed to develop a positive preference. If anything, on the second and third days, several individuals appeared to consistently avoid or escape from their laser-corner. The potential avoidance by these D2 ChR2 individuals appeared to grow with repetition, especially from the second to the third test days. Strikingly, these D2 laser-avoiders had previously shown positive self-stimulation in the spout task, suggesting that the motivational valence of their NAc D2 ChR2 stimulation had in a sense flipped from one situation to another.

If so, what explains the shift from positive self-stimulation to failure, or even to negative, for D2 ChR2 stimulation in NAc? One answer might be the relatively longer duration of laser stimulation in the location task than in the spout task. D2 ChR2 mice rarely received more than a single 1-sec NAc laser stimulation at a time in the spout task, whereas in the location task they often received two to four consecutive 1-sec bins of laser stimulation while remaining in the laser-corner. Conceivably, the valence of D2 ChR2 laser stimulations in NAc might shift from positive to neutral or even to negative as the duration persists for longer than 1 sec. Preference for shorter over longer durations has been reported for optogenetic self-stimulation of glutamatergic neurons in ventral tegmentum (<5 sec duration) [43]. Decades ago, rats were reported to prefer shorter 0.5–1 sec durations of electrical self-stimulation in lateral hypothalamus over longer >2 sec durations (<2 sec durations) [44]. A second alternative explanation could be due to stimulus/response differences between tasks. Active touch on a spout allowed stimulation to be instrumentally controlled by voluntary initiation of each new response and allowed stimulus attribution of laser to the associated laser spout or the act of touching it. By contrast, the location corner may have presented a more diffuse spatial context associated with laser, where laser could temporally also continue even if the rat only remained passively within. Further, the corner could be encountered inadvertently while running ‘laps’ around the chamber periphery, and so perhaps less readily perceived as controlled by an instrumental response. Finally, although order effects or additional virus incubation conceivably could be relevant because the spout-touch task was run a week before the location-based task, that seems unlikely because D2 ChR2 individuals reverted to positive self-stimulation again when retested on the spout task even after the location task.

Whatever the explanation, shifts or reversals of valence for D2 ChR2 stimulation in NAc suggest that the D2 NAc motivational role is not as reliably fixed as appetitve as D1 ChR2 stimulation role, but rather D2 can be relatively plastic in motivational valence. That is, D2 NAc stimulation can take on either positive, neutral or negative motivational valence depending situational factors, whereas D1 NAc stimulation remains more robustly positive across situations.

### Comparison to other NAc studies on D1 vs D2 roles

Our finding that D1 MSNs in NAc supported strong self-stimulation behavior here is consistent with many previous reports of D1 NAc participation in appetitive motivation and reward. For example, D1 ChR2 stimulation in NAc enhances seeking of drug reward [23, 44], and D1 pharmacological stimulation in NAc similarly amplifies incentive motivation to obtain or consume food, sex, drugs and other rewards [27,29–31,33]. Similarly, in dorsomedial neostriatum, optogenetic excitation of D1 MSNs supports robust self-stimulation on a laser-spout task [21]].

Conversely, the lack of positive role, and even negative-avoidance role, for NAc D2 neuron stimulation in the location task is consistent with a report that optogenetic D2 ChR2 stimulation in dorsomedial neostriatum is also avoided by mice [21]. An escape-avoidance role for D2 excitation in NAc is also consistent with reports that either optogenetic D2 NAc stimulation or neurochemical D2 receptor activation can suppress motivation to seek rewards [24,25,45,46]], or even be needed for negatively-valenced fearful threat reactions such as conditioned freezing or unconditioned anti-predator and escape behaviors [33,47].

Further, an appetitive-suppression or aversive role for D2 NAc excitation is also consistent with the ‘appetitive-NAc-inhibition’ hypothesis of reward, which posits hyperpolarization (rather than depolarization) of MSNs in NAc to be a primary mechanism for generating intense appetitive motivation [48–50]. By this hypothesis, NAc inhibition halts GABA release from NAc output projection axons, and so disinhibits downstream targets into relative excitation in hypothalamus, VP, and VTA. While a strong version of this hypothesis might predict that both D1 and D2 NAc inhibitions would be more effective at generating appetitive motivation than NAc excitations, a popular version holds that NAc D2 MSN inhibition especially contributes to appetitive motivation [51]]. By that view, pharmacological D2 agonist stimulations that promote reward seeking, such as D2/D3 agonist medication induction of addictive-like motivations in Parkinson’s patients with Dopamine Dysregulation Syndrome [52], would be seen as inducing neuronal inhibitions of D2 neurons via inhibitory G_i_ G-protein receptor mechanisms. The same explanation could be applied to virally-mediated increase in NAc D2 receptor expression that is reported to promote incentive motivation for food reward [35]. Conversely, pharmacological D2 receptor blockade by neuroleptic drugs, has long been known to reduce appetitive motivation for rewards [15,29,53–59]. Such D2 antagonists would be viewed as disinhibiting D2 neurons into relative excitation and GABA release by blocking the same G_i_ G-protein receptors.

By contrast, our results here in the spout-touch task indicate that direct neuronal excitation of D2 neurons in NAc can also be sufficient to generate positive self-stimulation, at least under some circumstances. Findings of an appetitive D2 role in NAc for ChR2 self-stimulation may be more surprising, yet is also consistent with some other studies. For example, optogenetic stimulation of D2 neurons in NAc also has been reported to amplify appetitive motivation, expressed as breakpoint effort to obtain food reward [34]]. Further, specific inhibition of D2 neurons in lateral neostriatum is reported to reduce motivation for reward similarly to inhibition of D1 neurons [23]. Finally, optogenetic excitation of D2 neurons in lateral neostriatum has been reported to support active self-stimulation behavior [22], similarly to our finding of NAc D2 self-stimulation. However, the authors interpreted lateral neostriatal D2 self-stimulation as sensorimotor in nature rather than motivational and suggested it to reflect a stimulus-response habit that merely became motorically stamped in by laser reinforcements. However, we note that our D2 ChR2 mice were not motorically rigid, but rather were flexible in altering response to pursue their laser spout when it moved to new locations (within 10 min for its final shift). They succeeded in tracking even when the positions of laser spout and non-laser spout were reversed from their initial locations, providing perfect stimulus opportunity to ‘habitually’ touch the now-inactive spout in the original laser location. Flexible following of a laser source seems to rule out a simple stimulus-response habit interpretation of NAc D2 self-stimulation, which would be expected to produce habits that more rigidly persevere, as insensitive to the shift in act-outcome relation. We conclude that our D2 ChR2 mice were genuinely motivated to seek NAc D2 neuronal stimulation.

Given that NAc D2 neuronal excitation can contribute to reward-related motivation, how can this positive appetitive role be explained? One potential explanation is that D2 neuronal activation in NAc recruited a nearly identical pattern as D1 NAc stimulation of distant Fos recruitment in other limbic structures, including ventral pallidum, lateral hypothalamus, ventral tegmentum, amygdala, hippocampal subiculum, and medial prefrontal cortex. Overall, there was about an 85% overlap between NAc D1-elicited and D2-elicited increases in Fos across these structures. Similar patterns of neurobiological activation in reward circuitry implies the possibility of similar psychological effects on incentive motivation. As a potential underlying basis, NAc D2 MSNs and D1 MSNs are recognized to both send ‘indirect’ output projections targets to nearly the same sites in ventral pallidum and lateral hypothalamus [13,14,16]. However, there were also differences in Fos evoked by D1 ChR2 versus D2 ChR2 stimulations, especially evident in the local Fos plumes surrounding optic fiber sites in NAc. Not only would different neurons be expected to be stimulated in D1 and D2 mice near the illuminated fiber tip (e.g., D1-expressing MSNs versus D2-expressing MSNs plus cholinergic interneurons), but activated neurons also differed in the degree or intensity of stimulated Fos expression (measured by the number of Fos-positive neurons counted within 50-micron square sampling boxes). D2 ChR2 laser stimulation produced less intense Fos plumes in NAc than D1 ChR2 stimulations, although the total size or diameter was similar for D1 and D2 plumes. D1 ChR2 stimulation generated a zone of 150% Fos elevation that extended 0.44mm across the fiber tip, and a further zone of >125% elevation that extended up to 0.84mm, and an outer plume of 110% elevation that spread throughout the full 1.06 mm diameter of the detectable plume. By contrast, in D2 Fos plumes the >150% elevation zone extended only 0.18 mm in diameter (less than half of the corresponding D1 plume zone), and the D2 >125% elevation zone had only a 0.56 mm diameter (two-thirds of the corresponding D1 plume zone). A mildest 110% plume, zone filled the 0.78mm outer diameter of D2 plumes, roughly three-fourths that of the D1 ChR2 outer plume. Further, diameters of viral spread were similar between D1 and D2 ChR2 mice (~0.9mm vs 0.8mm), any difference in Fos does not seem simply due to differences in infection. Less intense Fos elevation within a plume, suggests that fewer NAc neurons at any point within the equal-sized Fos plumes were stimulated by local laser to produce Fos in D2 ChR2 mice than in D1 ChR2 mice (as well as different local neurons in D1 vs D2 mice). Such neurobiological differences could contribute to behavioral differences in self-stimulation efficacy. However, despite these differences in local Fos plumes in D1 and D2 mice, similar levels of distant Fos were nonetheless recruited in other limbic structures, as described above. That suggests the possibility that D2 NAc excitation may actually have been more effective or efficient than D1 NAc excitation at recruiting functional connectivity and Fos production in target brain structures. That is, fewer D2 NAc neurons may need to be activated than D1 NAc neurons to produce the same degree of Fos activation in downstream networks. Alternatively, it is also conceivable that a ceiling exists for Fos increases in distant structures triggered by NAc stimulation, which was reached here in both D1 and D2 mice. If so, D1 ChR2 stimulation may have been unable to raise distant Fos further in other structures than D2 ChR2 stimulation, despite being more effective at generating intense Fos plumes in NAc and at supporting behavioral self-stimulation.

Overlap between D1 and D2 self-stimulation here may also reflect neuronal overlap, in that up to one-third of neurons in NAc shell have been suggested to express both D1 and D2 receptors together on the same MSN [13,17,60]. Any D1/D2 co-expressing subpopulation in NAc would likely have been activated in both D1 ChR2 mice and D2 ChR2 mice. That shared subpopulation of MSNs would potentially contribute to overlap in functional connectivity and in behavioral effects. Finally, we note that D2 receptors are expressed also by >80% of acetylcholine striatal interneurons [19,20], which also would have been excited by ChR2 stimulation in NAc of D2-Cre mice. NAc acetylcholine neurons are reported to contribute to appetitive motivation [61–63], and so laser excitation of NAc acetylcholine neurons could conceivably have contributed to D2 motivation effects underlying behavioral self-stimulation. The power of this explanation is constrained somewhat by the consideration that acetylcholine neurons also should have been stimulated by our location-based task, as well optogenetic D2 ChR2 stimulation in studies of neostriatum, including striatal regions that apparently do not support D2 self-stimulation, such as dorsolateral neostriatum [21]. However, acetylcholine neurons might play different motivation roles in different striatal regions, or even in different situations for the same NAc region, so this possibility still remains open.

## Conclusion

In conclusion, these findings indicate that D2 neuronal excitation in NAc can support moderate appetitive motivation to self-stimulate, at least under some conditions. By comparison, D1 MSN excitation in NAc supports more intense appetitive self-stimulation behavior, and remains reliably positive across multiple situations, including those where D2 neuronal excitation fails. Beyond NAc D2 contributing less robustly than D1 to appetitive motivation, D2 NAc excitation also appears capable of flexible shifts in motivational valence from positive to neutral, or even from positive to negative, in the same individuals as situations change. This suggests that D2 NAc roles in motivation may be ambivalent or plastic in valence, whereas D1 roles remain more distinctly positive. These results underline the complexity of D1 versus D2 neuronal functions in NAc, and add to evidence that NAc D1 and D2 neurons play distinct, yet potentially overlapping, roles in appetitive motivation.

## Supporting information

S1 File D1 and D2 ChR2 spout self-stimulation. Video shows side by side comparisons of spout-based self-stimulation in D1 and D2 ChR2 mice. **(Left)** D1 ChR2 mice approach and show robust self-stimulation behavior, using mouth and forepaws to earn 1mW, 1s, constant laser illuminations. **(Right)** D2 ChR2 mice also seek out laser-paired bottle spouts and self-stimulate, though at lower rates than D1 ChR2 mice.
(MOV)
